# Overfeeding Extends the Period of Annual Cyclicity but Increases the Risk of Early Embryonic Death in Shetland Pony Mares

**DOI:** 10.3390/ani11020361

**Published:** 2021-02-01

**Authors:** Nicky M. M. D’Fonseca, Charlotte M. E. Gibson, Iris Hummel, David A. van Doorn, Ellen Roelfsema, Tom A. E. Stout, Jan van den Broek, Marta de Ruijter-Villani

**Affiliations:** 1 Department of Clinical Sciences, Faculty of Veterinary Medicine, Utrecht University, Yalelaan 112, 3584 CM Utrecht, The Netherlands; chrlt.gibson@gmail.com (C.M.E.G.); irishummel1@gmail.com (I.H.); d.a.vandoorn1@uu.nl (D.A.v.D.); e.roelfsema@uu.nl (E.R.); t.a.e.stout@uu.nl (T.A.E.S.); M.Villani@uu.nl (M.d.R.-V.); 2 Department of Population Health Sciences, Faculty of Veterinary Medicine, Utrecht University, Yalelaan 7, 3584 CL Utrecht, The Netherlands; j.vandenbroek@uu.nl

**Keywords:** obesity, insulin dysregulation, hemorrhagic anovulatory follicle, anestrus, embryonic death

## Abstract

**Simple Summary:**

Obesity has been associated with altered reproductive activity in mares and may negatively affect fertility. To examine the influence of long-term high-energy (HE) feeding on fertility, Shetland pony mares were fed a diet containing 200% of net energy (NE) requirements during a three-year study. The incidence of hemorrhagic anovulatory follicles (HAF) and annual duration of cyclicity were compared to those in control mares receiving a maintenance diet. Day-7 embryos were flushed and transferred between donor and recipient mares from both groups; the resulting conceptuses were collected 21 days after transfer to assess conceptus development. HE mares became obese, and embryos recovered from HE mares were more likely to succumb to early embryonic death. The period of annual cyclicity was extended in HE compared to control mares in all years. The incidence of HAFs did not consistently differ between HE and control mares. No differences in embryo morphometric parameters were apparent. In conclusion, consuming a HE diet extended the duration of cyclicity, and appeared to increase the likelihood of embryos undergoing early embryonic death following embryo transfer.

**Abstract:**

Obesity has been associated with altered reproductive activity in mares, and may negatively affect fertility. To examine the influence of long-term high-energy (HE) feeding on fertility, Shetland pony mares were fed a diet containing 200% of net energy (NE) requirements during a three-year study. The incidence of hemorrhagic anovulatory follicles (HAF) and annual duration of cyclicity were compared to those in control mares receiving a maintenance diet. Day-7 embryos were flushed and transferred between donor and recipient mares from both groups; the resulting conceptuses were collected 21 days after transfer to assess conceptus development. HE mares became obese, and embryos recovered from HE mares were more likely to succumb to early embryonic death. The period of annual cyclicity was extended in HE compared to control mares in all years. The incidence of HAFs did not consistently differ between HE and control mares. No differences in embryo morphometric parameters were apparent. In conclusion, consuming a HE diet extended the duration of cyclicity, and appeared to increase the likelihood of embryos undergoing early embryonic death following embryo transfer.

## 1. Introduction

Obesity is an increasingly important health and welfare issue in horses, predisposing them to serious medical conditions such as insulin dysregulation [1], laminitis [2], and cardiovascular changes including myocardial hypertrophy [3], which collectively constitute the equine metabolic syndrome (EMS) [4]. Obesity has also been associated with altered reproductive cyclicity in mares, and is commonly believed to be detrimental to fertility [5]. However, whether and how obesity affects fertility in mares has yet to be explored objectively.

Ovulatory activity in mares is regulated primarily by changes in photoperiod. The physiological reproductive season begins in the spring, when the number of daylight hours increases, and ovulatory activity ceases again during late autumn and winter, when daylength shortens [6,7]. The duration of the period in which mares show ovulatory activity is an important potential contributor to fertility, because a larger number of normal estrous cycles equates to more opportunities for mating or insemination and fertilization. Body condition has also been shown to influence the regulation of ovulatory activity in mares and can, at least in part, override the influence of daylength [8,9]. Mares in good body condition show an abbreviated period of winter ovulatory inactivity or do not enter winter anestrus at all, which indicates a possible relationship between body condition and the regulation of cyclicity [8].

Obesity has also been suggested to increase the likelihood of mares developing a hemorrhagic anovulatory follicle (HAF) instead of undergoing rupture of the dominant follicle [5]. HAF formation reduces fertility primarily because it results in failure to release an oocyte [10]. In addition, it can lead to the development of a persistent luteal structure that markedly extends the inter-ovulatory interval [11], which presumably explains why Vick et al. [5] observed that obese mares (body condition score (BCS) > 7) [12] were more likely to exhibit extended periods (37–78 days) of continuously elevated circulating progesterone concentrations than non-obese feed-restricted mares. In a follow-up experiment examining the effect of metformin treatment on reproductive activity in obese mares, Vick et al. [5] observed extended periods of elevated progesterone concentrations associated with large persistent anovulatory follicles in both the metformin-treated and control groups. They concluded that mechanisms involved in triggering ovulation might be suppressed in obese mares, however the potential link between obesity and HAF formation in mares has yet to be confirmed.

In addition to the proposed effects on annual cyclicity and ovulation, obesity has been reported to reduce oocyte quality and alter embryonic development in women [13]. Overweight or obese women have significantly lower clinical pregnancy and live birth rates and a significantly higher risk of miscarriage following in vitro fertilization (IVF), compared to women of normal body-mass index [13]. Dietary composition has also been shown to affect embryonic development. Mice fed a high-fat diet showed lower oocyte quality and IVF success rates than mice fed a control diet [14]. Furthermore, the fetuses in mice fed a high-fat diet grew more rapidly, which has been proposed to increase the risk of the offspring developing metabolic syndrome later in life [15].

This study focused on the effect of chronic overfeeding on fertility and embryonic development in Shetland pony mares. Obesity was induced by long-term provision of a diet containing 200% of net energy (NE) requirements, and the incidence of HAF formation and annual duration of reproductive cyclicity were compared to mares fed a control diet (100% NE requirements). The effects on early embryonic development and quality were examined by flushing and transferring day 7 embryos between control and high-energy (HE) mares, to further try to examine at what stage embryo viability might be compromised. It was hypothesized that consumption of a HE diet would increase BCS and the incidence of HAF formation, extend the annual period of cyclicity, and compromise early embryonic development, leading to a reduced pregnancy rate and alterations in embryo morphometry.

## 2. Materials and Methods

### 2.1. Horses and Husbandry

**Figure 1:**
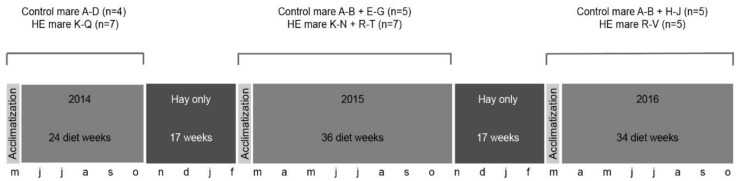
Organization of the feeding periods during study years 2014 to 2016, including the acclimatization periods (i.e., a one week period during which the high-energy (HE) diet was built up gradually to the full ration), the number of weeks during which the diet was fed and the hay-only periods in between, with the corresponding months. The ponies in the control (maintenance diet) and high-energy (HE; diet equating to 200% of net energy requirements) groups are described per study year.

The study was executed over a three-year period between February 2014 and October 2016. Twenty-two Shetland pony mares were assigned to either the control (*n* = 10) or HE groups (*n* = 12). Eleven Shetland pony mares (seven HE ponies (mean ± SD age, 4.6 ± 1.6 years) and four control ponies (mean ± SD age, 4.5 ± 1.3 years)) were recruited in 2014. At the time of recruitment, all mares had a moderate body condition score (range, 4–6/9) [12]. The feeding protocol started with one week of acclimatization, in which the HE diet was built up gradually to the full concentrate ration. Mares received their experimental diet for 24 weeks during the physiological breeding season of 2014; this was followed by a 17 week period of a hay-only diet during the winter. The HE diet was resumed in 2015 for a further 36 weeks, during which seven HE ponies and five control ponies were monitored (4/7 HE and 2/4 control ponies continued; 3 HE and 3 control ponies were added to the study (mean ± SD age, 5.0 ± 2.4 years)). This feeding period was again followed by a 17 week winter period of a hay-only diet. The HE diet was resumed for a third period of 34 weeks in 2016, during which five control and five HE ponies were monitored (3/7 HE and 2/5 control ponies continued; two HE and three control ponies were added to the study (mean ± SD age, 4.2 ± 1.3 years)). Overall, two control mares were retained throughout the entire three year period of the study, one control and seven HE mares were retained for two consecutive years, and seven control and five HE mares were included for only one year (Figure 1). The general health of all mares was assessed daily by monitoring heart rate, rectal temperature, and gait.

During the first year (2014), control ponies were housed as a group but fed individually, whereas HE ponies were housed and fed individually. Both groups were bedded on wood shavings. In between the experimental periods of 2014–2015 and 2015–2016 (winter break), the ponies were housed together as a single group in a straw bedded yard. During the second and third years (2015 and 2016), all ponies were housed and fed individually and bedded on shavings. Groups were allowed access to a sand paddock every other day to enable limited exercise and social contact throughout the study. Ethical approval for the study was granted by Utrecht University’s Committee for Animal Welfare (DEC 2014.III.01.004).

### 2.2. Diet

The composition of the diet has been described previously [16]. In brief, the diet of both the control and HE groups consisted of a concentrate feed (36% sugar + starch, 13% fat), grass hay (9% sugar, 2% fat) and a feed supplement to ensure adequate provision of minerals, trace elements and vitamins (Pavo Vital Complete; Pavo, Boxmeer, the Netherlands). Control mares were fed 100% of their daily NE requirements (85% in the form of hay and 15% as concentrate) as specified by the Centraal Veevoederbureau [17] (approximately 0.348 megajoule NE × BW^0.75^) to maintain a moderate body condition throughout the study. Mares in the HE group were fed 200% of their NE requirements (42.5% in the form of hay and 57.5% as concentrate) to induce weight gain, as previously described by Carter et al. (2009) with minor modifications [18]. Energy intake was adapted to weight gain throughout the experiment to maintain the 200% NE intake. During the winter periods, all mares were fed a hay-only diet of at least 100% NE requirements. All mares had free access to water and a salt lick (KNZ^®^; Hengelo, the Netherlands). Hay and concentrate were fed in multiple meals per day at 08:00, 13:00, and 17:00 h.

As reported in a previous article, the HE mares showed insulin dysregulation (ID) after 10–12 weeks of overfeeding in 2014, as determined by periodic oral glucose tolerance tests [16]. During the winter period when the HE diet was stopped, the ID was reversed. However, ID returned after 9–13 weeks of overfeeding in study year 2015. In short, ponies in the HE group became insulin dysregulated after 9–13 weeks of each overfeeding period.

### 2.3. Measurements

#### 2.3.1. Assessment of Obesity

All ponies were weighed weekly using a calibrated scale (Epelsa BCN100M: Grupo Epelsa, Madrid, Spain) to monitor weight gain. The BCS of each pony was assessed at the start and end of all three study years, using a 9-point scale [12].

#### 2.3.2. Hemorrhagic Anovulatory Follicles

The incidence of HAFs was detected by transrectal palpation and ultrasonography, using an ultrasound scanner (MyLab30, Esaote, the Netherlands) equipped with a 7.5-MHz linear-array transducer. Inclusion criteria for HAFs were described previously [19]. Mares had to initially show clear signs of estrus (i.e., uterine edema and cervical relaxation). Development of a HAF was suspected when the dominant follicle began to show increasing numbers of echoic specks, and was confirmed by failure of the follicle to evacuate but instead to continue growing and producing more echogenic particles, which eventually filled the whole follicle and then clotted to form a fluctuating cobweb-like internal structure. Finally, the HAF would further organize to form an abnormally large CH/CL-like structure and the mare would enter diestrus characterized by loss of endometrial edema and increased uterine and cervical tone. Examinations were performed once a week during the anestrous period, and at least twice a week after the first ovulation of the year. Once a dominant follicle exceeded 30 mm in diameter, examinations were performed daily until ovulation or formation of a HAF was detected. To aid reproductive synchronization, mares were treated with a PGF2α analogue (d-Cloprostonol: Genestranvet ^®^ 37.5 µg im, aniMedica GmbH, Senden-Bösensell, Germany) in the diestrous period following a normal ovulation or a HAF (at least 10 days after the HAF contents had clotted/organized). The number of estrous periods ending with normal ovulation or with formation of a HAF was monitored.

#### 2.3.3. Duration of Annual Cyclicity

The duration of annual cyclicity was determined by calculating the number of days between the first and last detected ovulation per study year for each individual mare.

#### 2.3.4. Embryonic Development

To examine the effect of the HE diet on early embryonic development, embryos were recovered from and transferred between control and HE mares as described below. During early estrus, mares were examined by ultrasonography every other day. Once a dominant follicle exceeded 30 mm in diameter, mares were examined daily until ovulation. When the dominant follicle exceeded 35 mm in diameter, donor mares were inseminated with fresh semen (minimum of 500 × 10^6^ sperm cells) from a single fertile stallion. Insemination was repeated every two days until ovulation. Recipient mares followed a similar monitoring schedule but were not inseminated. To aid synchronization between donors and recipients, mares were strategically injected with d-cloprostenol (Genestranvet^®^) during diestrus to induce luteolysis, and hCG (Chorulon 1000 IE iv, Intervet Nederland B.V., Boxmeer, the Netherlands) when they were in estrus with a pre-ovulatory follicle to induce ovulation. On day 7 after ovulation, the embryo was collected by uterine lavage with 3 × 1 L Lactated Ringer’s solution (supplemented with 0.5% [*v*/*v*] heat-inactivated fetal calf serum) as described previously [20]. After washing, recovered embryos were placed in holding medium (Syngro, Bioniche Inc.), their diameter was measured and their stage of development and quality were assessed [21]. Only good quality embryos (grade 1 or 2) were transferred to recipient mares that had ovulated between 0 and 2 days after the donor mare. After transfer, recipient mares were examined for pregnancy by transrectal ultrasonography 7 (ET + 7), 14 (ET + 14), and 21 (ET + 21) days after embryo transfer (ET); during these examinations the diameter and area of the embryonic vesicle were measured by examination of ultrasound images using Image J Fiji [22]. At ET + 21 (day 28 of gestation) the conceptuses were recovered under endoscopic guidance after puncture of the membranes and aspiration of the yolk sac and allantoic fluids, as described previously [23]. After recovery, day 28 conceptuses were washed repeatedly with sterile NaCl. The yolk sac and allantochorion were microscopically separated from the embryonic body using microsurgical scissors [23], and the embryonic body was measured (crown-rump length) and weighed.

Embryo transfers were performed as follows:

- Control donor to control recipient mare (C-C)
- HE donor to HE recipient mare (HE-HE)
- Control donor to HE recipient mare (C-HE)
- HE donor to control recipient mare (HE-C)

### 2.4. Statistical Analysis

Statistical analysis was performed using IBM SPSS Statistics for Windows, version 26 (IBM Corp., Armonk, NY, USA), and RStudio software (PBC, Boston, MA, USA). For all data, normality of distribution was confirmed by visual inspection of the Q-Q plot of the residuals. A linear mixed model with pony as a random effect, and year and group as fixed effects, was performed to compare the duration of annual cyclicity between control and HE mares. To compare the incidence of HAF formation between control and HE mares, the number of HAFs as a proportion of the total number of cycles (grouped binomial data) per pony was analyzed using a logistic regression with group, year and their interaction as independent variables. To compare the embryo recovery rate on day 7 after ovulation between control and HE mares, a logistic regression was performed using donor mare as a random effect and group as a fixed effect. The diameter of the conceptus on day 7 was compared between the control and HE groups using a linear mixed model with donor mare as a random, and group as a fixed, effect. Differences between groups with regard to the size of the embryonic body at ET + 14 and crown-rump length and weight of the embryo after recovery were analyzed using a linear model with group as an independent effect. To assess differences between groups in diameter of the conceptus vesicle and area of the vesicle at ET + 7, ET + 14, and ET + 21 for pregnancies that were maintained until day 28 of gestation, a linear mixed model was used with recipient mare as a random, and group, day (age of the embryo in days) and the interaction group-by-day as fixed effects. To compare the ongoing-pregnancy rate after embryo transfer (day 28 of gestation) between control and HE donor mares, an exact confidence interval for the difference of the two proportions was calculated as previously described [24]. For all analyses, Akaike’s Information criterion (AIC) was used for model reduction, with the model with the lowest AIC considered the most appropriate. For the effects in the final model, 95% profile (log) likelihood confidence intervals were calculated. All data were presented as mean ± SD.

## 3. Results

### 3.1. Obesity

Mean ± SD BCS and body weight (BW) for the control and HE groups are presented for the start and end of each study year in Table 1. Mean BW of the HE group increased by 27% during year 1, by 30% during study year 2, and by 26% during study year 3. For HE ponies retained into a second year, BCS and BW did not return to starting values following a winter period at around maintenance diet. Mean BW of the control group increased by 7% during study year 1, decreased by 6% during study year 2, and did not change in year 3. After 2 successive years of consuming a HE diet, overfed ponies exhibited a slightly stiff gait and, on a hard floor, walked with short strides, raising the suspicion of subclinical laminitis; this was further evaluated and described in a related study [25].

### 3.2. Hemorrhagic Anovulatory Follicles

The total and mean ± SD number of HAFs and cycles, and the percentage of HAFs over the total number of cycles are presented for the control and HE group per study year in Table 2. The data showed a year-group interaction, revealing an effect on the incidence of HAFs for control versus HE mares in years 1 (95% Confidence Interval (1.16, 17.90); Odds Ratio 3.89), 2 (95% CI (0.039, 0.55); OR 0.16) and 3 (95% CI (0.55, 5.90); OR 1.69); however, HE mares had a higher mean number of HAFs in years 1 and 3, and a lower number in year 2, compared to controls.

### 3.3. Duration of Annual Cyclicity

**Figure 2:**
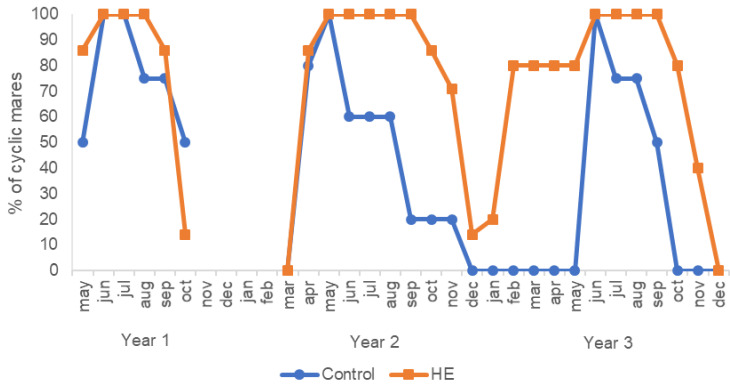
Percentage of cyclic mares per month for the control (maintenance diet) and high-energy (HE; diet equating to 200% of net energy requirements) groups during study years 2014–2016 (years 1–3). Measurements were stopped from November 2014 until March 2015. Year 1: *n* = 4 control and 7 HE mares; Year 2: *n* = 5 control and 7 HE mares; Year 3: *n* = 4 control and 5 HE mares.

Three of four control mares and two of seven HE mares had entered transitional anestrus when measurements were stopped in year 1, whereas in years 2 and 3 measurements were stopped only when all mares had entered the transitional phase (Figure 2). The data showed a year-group interaction, revealing a substantial difference in the duration of annual cyclicity for control versus HE mares in years 1 (95% CI (−108.24, −6.75)), 2 (95% CI (−141.96, −46.61)), and 3 (95% CI (−203.54, −94.35)). Mean duration of cyclicity was longer for HE mares (year 1: 118 ± 27 days; year 2: 208 ± 37 days; year 3: 239 ± 57 days) than control mares (year 1: 82 ± 31 days; year 2: 103 ± 76 days; year 3: 75 ± 19 days).

### 3.4. Embryonic Development

#### 3.4.1. Embryo Recovery and Pregnancy Rates

Mares were inseminated at a total of 93 cycles over the three study years. No group effect was found for the embryo recovery rate at day 7 after ovulation (control mares: 24/33 (72.7%) cycles yielded an embryo; HE mares: 40/60 (66.7%) cycles yielded an embryo). Twenty-six embryos were transferred to recipient mares, of which five did not result in a detectable pregnancy at day ET + 7 (day 14 of gestation: 81% pregnancy rate), while two pregnancies were lost between ET + 7 and ET + 14 (day 21 of gestation; 10% embryonic loss). The remaining flushed embryos were used for other investigations (immunofluorescent staining, PCR, and electron microscopy). Pregnancy rates at ET + 21 (day 28 of gestation) are presented per transfer group in Table 3. Overall, embryos originating from a HE donor mare were more likely to fail to reach day 28 of development (7/17; 41%), than embryos originating from a control mare (0/9: 0%), irrespective of the feeding regime of the recipient mare. Indeed, the 95% confidence interval (0.07843, 0.67076), although wide, does not cross 0 which strongly indicates that HE donor mares were more likely to yield embryos that failed to survive to day 28 than control donor mares.

#### 3.4.2. Diameter and Area of the Conceptus Vesicle

The mean diameter of the blastocyst was not different between control (255 ± 122 µm) and HE (298 ± 145 µm) mares on day 7 after ovulation (95% CI (−44.36, 149.71)). Mean ± SD values for the ultrasonographically determined diameter and area of the conceptus vesicle at ET + 7, ET + 14 and ET + 21 are presented per transfer group in Table 3. For both the area and diameter of the conceptus vesicle, the final model only contained ‘day’ as a fixed effect, and no group or group by day effects were found, i.e., there was no effect of either donor or recipient feeding status on conceptus size at any stage of development. The ‘day’ effect showed an overall difference between days 7, 14 and 21 after ET in diameter (ET + 7: 95% CI (11.57, 15.36); ET + 14: 95% CI (11.62, 16.44); ET + 21: 95% CI (15.84, 20.67)) and area (ET + 7: 95% CI (84.69, 212.45); ET + 14: 95% CI (308.68, 462.60); ET + 21: 95% CI (504.73, 659.16)) of the conceptus vesicle, i.e., there was an increase in area and diameter of the conceptus vesicle over time.

#### 3.4.3. Size and Weight of the Embryo Proper

Mean ± SD values for the length of the embryo proper at ET + 14 and the crown-rump length and weight of the embryo at ET + 21 are presented in Table 3. For the mean ultrasonographically measured length of the embryo proper at ET + 14, and crown-rump length and weight of the embryo at ET + 21, no group effect was found i.e., there was no effect of donor or recipient mare feeding status on size of the embryo proper.

## 4. Discussion

The aim of the present study was to determine the effect of chronic HE diet provision/overfeeding on fertility and embryonic development in Shetland pony mares. It was hypothesized that a long-term HE diet would induce obesity and increase the incidence of HAFs, extend the duration of annual cyclicity, and compromise early embryonic development or survival.

We did find a difference in the incidence of HAF formation between control and HE mares during all three study years. However, HE mares had a larger percentage of cycles ending with a HAF in years 1 and 3, and a lower percentage in year 2 compared to controls. This suggests that the occurrence of HAFs is more related to other differences between individuals than to energy level of the diet. This is contrary to the suggestion of Vick et al. [5], who reported a possible relationship between obesity and an increased incidence of persistent anovulatory follicles. It is possible that the difference in outcomes is in part because we looked at the incidence of HAFs as a percentage of the total number of cycles while Vick et al. [5] just reported the incidence of HAFs and periods of extended luteal activity. Alternatively, between study differences may be breed and/or age-related since Vick et al. [5] examined mixed light horse breed mares that were older (8 to 21 years) than the Shetland pony mares enrolled in our study (3 to 9 years). In general, differences in HAF formation between individual mares is characterized by the existence of so-called ‘repeaters’, i.e., mares with high HAF recurrence rates. Both the control and HE group contained individual mares with high HAF recurrence rates, which was not related to diet, and the relatively small total number of mares in the study may have allowed these individuals to influence the overall patterns observed. A previous study examining 721 mares over 1845 estrous cycles during a 5-year period found a HAF incidence of 8.2% [11]; moreover, 43.5% of mares that developed a HAF did so at least one more time during subsequent estrous cycles within the same breeding season [11]. The incidence of HAFs in that study significantly increased with mare age, with a 4.4% incidence for mares aged 6–10 years compared to 13.3% for mares aged 16–20 years. A second factor that could have influenced the incidence of HAF formation, and resulted in difference to Vick et al. [5], is the repeated use of prostaglandin F2α (PGF2α) analogues for induction of estrus. PGF2α analogue administration has been associated with a higher incidence of HAF formation in post-treatment cycles than after spontaneous return to estrus [26]. Mares forming HAFs at the end of PGF2α-induced estruses had higher LH concentrations 10 days post ovulation in the preceding spontaneous cycle, and it was suggested that elevated concentrations of LH may predispose to HAF formation [26]. During the present study, all mares were treated with a PGF2α analogue in the diestrus following a normal ovulation or HAF formation, with the exception of cycles in which they were used as an embryo transfer recipient; this could have influenced the likelihood of HAF formation in both groups.

As predicted, consuming a HE diet extended the duration of annual cyclicity in mares in all years. The extension of the period of cyclicity appears to be linked to diet-induced obesity, since all but two HE ponies developed obesity (BCS > 7: one pony in year 1 had a BCS of 5, and one pony in year 3 had a BCS of 5.5). Previous studies found a similar association between body condition and the duration of winter ovulatory inactivity [8,9], which was proposed to be related to the elevated plasma leptin concentrations observed in obese horses [27]. Indeed, it is thought that leptin acts as the primary messenger of metabolic (body condition) status in terms of central regulation of hormones involved in cyclicity [8,9]. However, since we did not measure plasma leptin concentrations in the present study, we were not able to confirm this association. In addition, it is possible that the differences would have been even more extreme if the HE mares had been overfed year round, rather than having the period of hay-only ‘recovery’ during the winter. In this respect, we are not able to definitively distinguish between the effects of obesity and increased energy availability as responsible for extending the period of cyclicity.

Another endocrine contributor to the increased duration of annual cyclicity is insulin and in particular the post-prandial peaks in insulin secretion [28]. In sheep, insulin has been suggested to influence reproductive activity by modulating GnRH secretion [29]. Similarly, in mares, the duration of annual cyclicity has been reported to be associated with the presence or absence of post-prandial insulin peaks, which differ depending on feed intake [8]. In a three-year study, seasonal anestrus was seen in 44% of well-fed (WF) mares (maintenance diet), whereas 80% of food restricted (R) mares (approximately 50% of energy requirements calculated on the mares’ body weights measured at the beginning of the experiment) showed seasonal anestrus during their first winter and 100% during their second and third winters. Twenty percent of mares fed a variable diet (fed either as the WF or R group, mimicking the normal seasonal variations of energy availability in a natural pasture) never showed winter ovulatory inactivity. Post-prandial plasma glucose and insulin concentrations increased significantly in the WF group, but not in food restricted (R) mares. Mares fed a variable diet had similar postprandial insulin peaks when fed the WF group diet, but not when fed the R group diet, demonstrating that the insulin response adapts to feed intake. In a previous publication describing the same ponies as the current study, we showed hyperinsulinemia during an oral glucose tolerance test in the HE but not the control mares [16], which indicates that the HE mares were in a hyperinsulinemic state at least three times a day after consuming the high concentrate load, with insulin concentrations remaining above reference limits for approximately four hours. Salazar-Ortiz et al. (2011) however suggested that insulin has no direct effect on reproduction in horses, since insulin concentrations adapt to the levels of feed intake and therefore provide a variable message, whereas the duration of winter inactivity is stable [8]. They proposed the effects of body condition and leptin to play a more important role [8]. However, the WF mares in the study of Salazar-Ortiz et al. (2011) were fed a maintenance diet, whereas the HE mares in our study were fed a double maintenance diet. The post-prandial insulin peaks in the HE mares in our study could therefore have been higher than those reported by Salazar-Ortiz et al. (2011), which might have influenced the duration of annual cyclicity. At this point however, no definitive conclusion can be drawn.

In the present study, embryos from HE donor mares were more likely to fail to establish pregnancy or to succumb to early embryonic death following embryo transfer than embryos from control donor mares, which suggests an association between a HE diet and/or obesity and embryo viability/developmental competence in ponies and is consistent with findings in women undergoing IVF [13]. Our findings suggest that deleterious effects of overfeeding on embryo development occur during the periconception period up to day seven post ovulation, since no pregnancy losses were seen when embryos from control donor mares were transferred, irrespective of the feeding status of the recipient mare. This finding further suggests that any influence of diet on the likelihood of successful pregnancy after embryo transfer in Shetland pony mares depends more on oocyte or early embryo quality than effects on the uterine environment. No effects of diet on day 28 conceptus or embryo morphology were evident, which is contrary to findings in mice [15]. However, these conclusions regarding embryo survival and growth should both be interpreted carefully since they are based on a small number of embryos/conceptuses, which might have respectively accentuated or masked any small differences.

The difference in embryo survival between control and HE donor mares could be explained by metabolic changes. The intrafollicular environment in mares has been shown to be altered by obesity and metabolic disease, as has been reported for other species [30,31]. Concentrations of insulin, leptin, and inflammatory cytokines in follicular fluid (FF) were highly correlated with those in serum and, therefore elevated in the follicles of mares with EMS compared to control mares [30]. Moreover, obese mares with decreased sensitivity to insulin show significantly higher FF concentrations of insulin and leptin than controls [31]. Besides the effect on preovulatory FF composition, adiposity has been shown to alter oocyte lipid content, and gene expression in granulosa and cumulus cells, of obese mares [31]. In cattle, exposing oocytes to insulin during in vitro maturation resulted in a decreased blastocyst formation rate and led to upregulation of genes involved in lipid metabolism in day 8 blastocysts, indicating that exposure of oocytes to insulin had an effect that lasted until at least the blastocyst stage [32]. Since the overfed mares in the present study were previously shown to exhibit hyperinsulinemia in response to an oral glucose challenge [16], it seems reasonable to propose that elevated plasma and FF insulin concentrations may have compromised oocyte and embryo developmental competence. However, follicular fluid insulin concentrations were not measured. A previous study also described the addition of leptin (10–100 ng/mL) during in vitro maturation of equine oocytes, which had beneficial effects for the success of meiotic maturation and fertilization after ICSI, but impaired embryonic development, with 100 ng/mL leptin reducing the percentage of the 2-cell stage embryos reaching the 4 to 8 cell stage [33]. In mice, elevated leptin concentrations have been shown to exert both positive and negative effects on embryo development in vitro, with the direction of the effect depending on the body condition of the oocyte donor [34]. Leptin had a beneficial effect on the development of embryos recovered from obese females, but a negative effect on embryos recovered from control females. In the current study, no effect of diet/body condition was seen on embryo recovery rates on day 7, but it is possible that increased intrafollicular concentrations of leptin in HE mares impaired embryo viability and predisposed to failure to establish pregnancy after transfer, or pregnancy loss at a later stage of gestation (before day 28), for embryos derived from HE mares.

## 5. Conclusions

In conclusion, obesity as a result of long-term feeding of a HE diet extended the period of annual cyclicity and increased the incidence of post embryo-transfer early pregnancy loss in Shetland pony mares, but did not appear to influence the formation of HAFs. Further research into the relationships between obesity-related metabolic and endocrine alterations and their effects on follicle development, the intrafollicular environment, and oocyte quality would further clarify the impact of nutritional status on female fertility.

## Figures and Tables

**Table 1:** Mean ± SD body condition score (BCS) and body weight (BW) for the control and high-energy (HE) groups for the start and end of each diet year (years 1–3).

Year		Mean ± SD BCS		Mean ± SD BW (Kg)	
		Control	HE	Control	HE
1	Start	4 ± 1	5 ± 1	152 ± 22	161 ± 28
	End	3 ± 1	8 ± 1	163 ± 23	205 ± 36
2	Start	6 ± 0	7 ± 1	176 ± 12	189 ± 35
	End	5 ± 1	9 ± 0	166 ± 8	245 ± 33
3	Start	5 ± 1	7 ± 1	186 ± 10	208 ± 13
	End	5 ± 1	8 ± 0	186 ± 12	263 ± 16

**Table 2:** Total and mean ± SD number of hemorrhagic anovulatory follicles (HAFs) and cycles, and the percentage of HAFs/cycles for the control and high-energy (HE) groups per diet year (years 1–3), with the corresponding number of mares per group.

Year	Number of Mares		Total HAFs		Total Cycles		Mean ± SD HAFs		Mean Cycles ± SD		Percentage HAFs/Cycles	
	Control	HE	Control	HE	Control	HE	Control	HE	Control	HE	Control	HE
1	4	7	3	20	24	56	1 ± 1	3 ± 2	6 ± 2	8 ± 1	13%	36%
2	5	7	8	4	32	80	2 ± 1	1 ± 1	6 ± 2	11 ± 1	25%	5%
3	5	5	5	12	29	46	1 ± 1	2 ± 3	6 ± 1	9 ± 1	17%	26%

**Table 3:** The pregnancy rate at day 28 of gestation (21 days after embryo transfer). Mean ± SD diameter of the conceptus and area of the embryonic vesicle at 7 (ET + 7), 14 (ET + 14), and 21 (ET + 21) days after embryo transfer (ET), size of the embryo at ET + 14 and crown-rump length and weight of the embryo at ET + 21, presented per embryo transfer group (control to control mare (C-C), control to high-energy mare (C-HE), high-energy to control mare (HE-C), and high-energy to high-energy mare (HE-HE)).

Parameter	Group of Transfer	ET + 7	ET + 14	ET + 21
Pregnancy rate at day 28 of gestation (day 21 after ET)	C-C	−	−	100% (4/4 successful pregnancies)
	HE-HE	−	−	56% (5/9 successful pregnancies)
	C-HE	−	−	100% (5/5 successful pregnancies)
	HE-C	−	−	63% (5/8 successful pregnancies)
Diameter of the conceptus (mm)	C-C	10.9 ± 2.5	28.6 ± 3.1	32.4 ± 3.6
	HE-HE	14.1 ± 1.6	25.4 ± 4.8	30.2 ± 1.7
	C-HE	13.1 ± 4.5	27.0 ± 3.8	31.1 ± 5.5
	HE-C	15.6 ± 2.7	29.1 ± 4.3	33.2 ± 5.0
Area of the vesicle (mm)	C-C	93.7 ± 43.4	570.9 ± 129.7	735.0 ± 149.5
	HE-HE	155.0 ± 36.7	449.7 ± 99.0	652.3 ± 87.1
	C-HE	146.7 ± 93.0	506.2 ± 152.0	738.8 ± 192.7
	HE-C	185.8 ± 61.1	609.8 ± 152.3	789.1 ± 186.8
Size of the embryo (mm)	C-C	−	3.29 ± 0.53	-
	HE-HE	−	3.46 ± 0.81	-
	C-HE	−	3.89 ± 0.84	-
	HE-C	−	3.60 ± 0.74	-
Crown-rump length (mm)	C-C	−	−	9.3 ± 0.3
	HE-HE	−	−	10.1± 1.5
	C-HE	−	−	9.7 ± 1.4
	HE-C	−	−	11 ± 0
Weight (mg)	C-C	−	−	162 ± 26
	HE-HE	−	−	182 ± 66
	C-HE	−	−	185 ± 63
	HE-C	−	−	170 ± 42

## Data Availability

The data presented in this study are available in Overfeeding Extends the Period of Annual Cyclicity but Increases the Risk of Early Embryonic Death in Shetland Pony Mares, D’Fonseca et al. 2021.
